# Acupuncture as Treatment of Hot Flashes and the Possible Role of Calcitonin Gene-Related Peptide

**DOI:** 10.1155/2012/579321

**Published:** 2011-10-26

**Authors:** Anna-Clara E. Spetz Holm, Jessica Frisk, Mats L. Hammar

**Affiliations:** ^1^Division of Obstetrics and Gynecology, Department of Clinical and Experimental Medicine, Faculty of Health Sciences, Linköping University, 581 85 Linköping, Sweden; ^2^Department of Obstetrics and Gynecology in Linköping, County Council of Östergötland, 581 85 Linköping, Sweden

## Abstract

The mechanisms behind hot flashes in menopausal women are not fully understood. The flashes in women are probably preceded by and actually initiated by a sudden downward shift in the set point for the core body temperature in the thermoregulatory center that is affected by sex steroids, **β**-endorphins, and other central neurotransmitters. Treatments that influence these factors may be expected to reduce hot flashes. Since therapy with sex steroids for hot flashes has appeared to cause a number of side effects and risks and women with hot flashes and breast cancer as well as men with prostate cancer and hot flashes are prevented from sex steroid therapy there is a great need for alternative therapies. Acupuncture affecting the opioid system has been suggested as an alternative treatment option for hot flashes in menopausal women and castrated men. The heat loss during hot flashes may be mediated by the potent vasodilator and sweat gland activator calcitonin gene-related peptide (CGRP) the concentration of which increases in plasma during flashes in menopausal women and, according to one study, in castrated men with flushes. There is also evidence for connections between the opioid system and the release of CGRP. In this paper we discuss acupuncture as a treatment alternative for hot flashes and the role of CGRP in this context.

## 1. Introduction

Hot flashes are classic menopausal symptoms in women [1, 2]. However, flashes are also reported by 43–77% of men after castration therapy [3–5], and they usually persist for many years and may impair quality of life [4]. Night sweats can be severe enough to cause sleep disturbance. In addition, hot flashes can be associated with anxiety, nausea, tachycardia, and tachypnea, as well as pressure in the head and chest. Hot flashes also occur in men with testicular insufficiency and in “normal aging men,” but to a lesser extent [6, 7]. Nearly all reported flashes in women and men after castration therapy are also objectively confirmed by increased cutaneous blood flow, skin conductance, and/or skin-temperature [8–11]. 

The physiology of hot flashes is not known in detail, but probably involves the core body temperature, neuromodulators, and peripheral vasculature and sweat glands. Vasomotor symptoms have been shown to correlate with the decrease in estrogen production during the menopausal transition and with testosterone decreases after castration therapy with orchiectomy or GnRH Analogues in men with prostate cancer. Estrogens, testosterone, and also other hormones are potent neuromodulators of the central nervous system [12]. Freedman and Subramanian have demonstrated that women with hot flashes have an increased core temperature and a reduced thermoneutral zone compared with women without flashes [13]. It is possible that fluctuations in estrogen concentrations cause changes in other hormones or neurotransmitters in the central nervous system that lead to an alteration in core temperature and a narrower thermoneutral zone in women predisposed to hot flashes [14]. A narrow thermoneutral zone causes reactions that lower central body temperature when the actual central temperature reaches or exceeds the upper limit of the neutral zone. Such reactions are vasodilation causing increased blood flow in the upper trunk, arms, and hands and irradiation of heat, that is, energy to the surrounding environment and activation of sweat glands, which leads to energy loss by evaporation. Another theory is that decreased endogenous estrogen concentrations also decrease hypothalamic *β*-endorphins that lead to an instability in the hypothalamic thermoregulatory centre [15, 16]. When the set point in the thermoregulatory center is suddenly lowered, reactions are initiated that decrease body temperature, acting in the same way as when the upper limit of the thermoneutral zone is exceeded. We suggest that testosterone plays a role in men similar to the role played by estrogen in women in this context and thus when testosterone concentrations are lowered as they are after castration therapy, thermoregulation becomes less stable. 

The relationship between serotonin and temperature control has long been recognized. Probably both noradrenalin and serotonin may affect the risk of hot flushes via a narrowed thermoneutral zone [13]. Serum levels of serotonin are lower in postmenopausal women than the levels found before menopause, and estrogen therapy has been shown to normalize these levels. Estrogen withdrawal causes a reduction in circulating serotonin, resulting in an upregulation of the 5-HT_2A_ receptor in the hypothalamus [17]. It has thus been suggested that both the concentrations of *β*-endorphins and serotonin in the hypothalamus decrease with decreasing estrogen concentration [18]. The reduced *β*-endorphin and serotonin concentrations increase the release of noradrenaline, and this may in turn cause sudden drops in the set point in the thermoregulatory centre in the hypothalamus and elicit inappropriate heat loss [18–21]. According to this hypothesis, any intervention that increases estrogen, *β*-endorphin, or serotonin concentrations or decreases noradrenalin levels may be expected to reduce hot flashes. 

The heat loss during the hot flash and sweating seems to be achieved by activation of cholinergic sweat glands and vasodilation of the skin, and these reactions may be mediated by the potent vasodilator calcitonin gene-related peptide (CGRP) [22]. Endogenous opioids modulate the release of the potent endothelium-dependent vasodilator CGRP at the spinal cord level [23, 24]. When CGRP is administrated intravenously to healthy male volunteers, it produces symptoms very similar to hot flashes, with a dose-dependent increase in cutaneous blood flow [25]. CGRP has been found to increase in plasma during hot flashes in postmenopausal women [26–29] and also, according to one study, in men with flashes who had been castrated due to carcinoma of the prostate [11]. Urinary excretion of CGRP over 24 h is higher in flashing postmenopausal women than in postmenopausal women without hot flashes, and in a group of postmenopausal women with hot flashes CGRP in 24 h urine decreased significantly after 12 weeks of successful treatment with acupuncture [30].

## 2. Treatment of Hot Flashes

The gold standard for treating hot flashes is estrogen therapy [12] which reduces the frequency and severity of hot flashes by 75% percent compared to placebo according to a meta-analysis from the Cochrane Database System [31]. Since hormonal treatment is at present controversial, largely as a result of results like those from the HERS study and the WHI study, there is a need for other nonhormonal treatment alternatives [32, 33]. Findings from these and other studies have led to more restrictive recommendations from the authorities on the use of estrogens and substantially decreased the use of hormone therapy. Therefore, many women today have climacteric symptoms including flashes but abstain from hormone replacement therapy. Furthermore, numerous women with breast cancer and men with prostate cancer have troublesome hot flashes but should not use sex steroid therapy because of the risk of cancer recurrence. As pointed out by, for example, Borrelli and Ernst, the potential serious side effects from hormone replacement therapy cause a great need for alternative and complementary treatments of hot flashes [34]. 

Progestagens have been shown to reduce hot flashes by 80–90% [35, 36], but their side effects include weight gain, fluid retention, and mastalgia [35] and should not be given to women with breast cancer.

Tibolone, a synthetic hormone that acts on sex hormone receptors, has also been shown to be as effective as estrogen therapy for treating hot flashes [37, 38]. Tibolone, however, should not be used in women who have had breast cancer because of the risk of recurrence and because it causes other side effects similar to those caused by estrogens [39]. 

Clonidine, selective serotonin reuptake inhibitors (SSRI), and gabapentin may decrease the frequency of hot flushes and the distress caused by them. The use of phytoestrogens and black cohorsh showed mixed results. The mechanism of action of SSRIs is thought to involve increased serotonin levels and thereby less severe vasomotor symptoms [40]. SSRIs reduce hot flashes by up to 50–60% compared to 80% reduction in women using estrogen [41]. However, recent data suggest that at least some SSRIs are associated with an increased risk of death from breast cancer, probably because SSRIs inhibit cytochrome P450 2DG (CYP2D6), which is necessary for the metabolism of tamoxifen, thus reducing the effect of the tamoxifen given to many women as part of treatment for breast cancer. Paroxetine is the one SSRI that is the strongest inhibitor of CYP2D6 and should therefore not be given to women with breast cancer [42]. 

Venlafaxine is the SSRI most frequently prescribed as an alternative to estrogens for the treatment of climacteric symptoms. In addition to its function as an SSRI, venlafaxine also acts as a noradrenergic reuptake inhibitor and is therefore called serotonin-noradrenaline reuptake inhibitor (SNRI). It is the most frequently prescribed alternative to estrogens [41] and halves the severity of hot flashes. Even if the effect is significant, it is only marginally better than the effect of placebo [43]. 

Lifestyle factors seem also to contribute to strengthening climacteric symptoms; greater BMI is a risk factor for hot flashes, as are smoking and high consumption of caffeine and alcohol [44]. Weight loss, regular exercise, and smoking cessation have also been recommended in order to decrease hot flashes. The efficacy of these recommendations has not been demonstrated, however, because of lack of sufficient clinical trials according to the Cochrane Database System Review [12, 45]. Physically active postmenopausal women have a lower occurrence of vasomotor symptoms, perhaps due to a higher central opioid activity [46, 47]. Acupuncture also seems to be effective in reducing the intensity and frequency of hot flashes in women and in men deprived of sex steroids due to prostate cancer [22, 48–53].

## 3. Acupuncture

Acupuncture is known as one of the oldest healing systems in the world and is a part of traditional chinese medicine (TCM). Variants of TCM acupuncture, based on the old Taoist theories of Yin and Yang and Qi, are practiced throughout the Western World today [54]. The physiological processes involved in acupuncture treatment are not fully known, but factors of importance may include changes in autonomic nerve functioning [55–57] and may affect hormones such as cortisol [58, 59], oxytocin [60, 61], neuropeptides as *β*-endorphin [62], serotonin [63, 64], and cytokines [65–67] and alterations in collagen network communication [68, 69]. Acupuncture probably affects serotonin and noradrenalin activity in the central nervous system [70, 71], and thus has the potential to influence the thermoregulatory centre, making it more stable [22]. Acupuncture may also have peripheral effects and cause the release of substance P, vasoactive intestinal peptide, and CGRP [72–74]. Probably the effects of acupuncture are caused by multicomponent, complex interventions. In sham-controlled studies, attempts are frequently made to control the needling effect by controlling the location, insertion depth, stimulation, needle size, and number. However, several other potentially therapeutic acupuncture-specific components may be present in the control group; these include nonspecific components (time, attention, credibility, and expectation) and specific non-needling components such as psychological history, diagnosis, and education and also physiological events like palpation and moxibustion [75].

One of the traditional forms of acupuncture is manual acupuncture (MA), where the needles are inserted in the specific acupuncture points, according to TCM. Often the needles are twirled to evoke the DeQui sensation, characterised by a distinct sensation of distension and numbness [62]. The DeQui sensation is believed to activate A-delta fibers from free nerve endings in the skin or from high-threshold ergoreceptors in the muscle. Electroacupuncture (EA) is derived from MA, with the addition of electric stimulation applied to one or two pairs of the needles, either applied with a high (80–100 Hz) or low frequency (2 Hz). EA has been shown to be more powerful than MA in studies on pain treatment [76, 77]. This stimulation is believed to activate peripheral nerve endings, muscles, and also connective tissue. The nerve stimulation causes afferent signals, which increase, for example, central *β*-endorphins, and serotonin and probably also activate receptors [78–80]. 

### 3.1. Acupuncture and Hot Flashes

Acupuncture has been tried for hot flashes since it seems to increase central *β*-endorphin activity [62], which would as a result make thermoregulation more stable and in turn decrease vasomotor symptoms [81, 82]. Some studies have shown a decreased activity, measured by fMRI, in the amygdala and hypothalamus, when acupuncture is given [83]. It may be speculated that during an incident of hot flashes there is a high neuronal activity in the hypothalamus and that acupuncture may reduce this activity, perhaps mediated by increased *β*-endorphin release and decreased noradrenalin activity.

Acupuncture has been found to decrease the number of hot flashes by at least 50% but does not seem to be as effective as estrogen therapy [84]. EA has been associated with a decreased number and intensity of hot flashes in menopausal women, both with and without breast cancer [22, 49–52], and also in men treated by castration due to prostate cancer [48, 53]. While pharmacological studies often use “placebo pills” for treatment of the control groups, it has been more problematic to find a credible but still inert sham technique that may be used in acupuncture studies. Several devices have been tried [85, 86], but these methods do not seem to be totally without effect, probably because they cause neuronal stimulation attributable to local pressure on or beside acupuncture points and induce tactile neuronal stimulation [87, 88]. Earlier studies have shown a better effect of EA on pain in lateral epicondylalgia [77] and low back pain [76] than with acupuncture using superficial needle insertion (SNI). Therefore, Wyon et al. [22] randomised women with hot flashes to either EA or superficial needle insertion (SNI) in the belief that EA would have a superior effect on hot flashes compared to SNI. It was not, however, possible to see any differences in effect of treatment, although, in the EA group, the reduction of hot flashes was sustained for a longer period of time after treatment than in the SNI group. Frisk et al. [53] also randomised between EA and MA in men castrated due to prostate cancer, but they were unable to show any differences between the treatment options regarding reduction in number of hot flashes.

In a systematic review article [89], the effectiveness of acupuncture versus sham acupuncture for treatment of hot flashes was assessed. They found six randomised clinical studies of acupuncture versus sham acupuncture but were not able to show any differences between the effects on hot flashes of acupuncture versus sham acupuncture. They concluded, however, that the sample was too small and that different types of sham-acupuncture were used in the different studies [89]. 

It is of course of great interest and importance to find out if acupuncture would affect other climacteric symptoms than the hit flashes. There are, however, to our knowledge no studies on the effects of acupuncture on, for example, excessive sweating, anxiety, or other climacteric disorders as primary outcome.

## 4. Calcitonin Gene-Related Peptide (CGRP)

CGRP is a 37-amino-acid neuropeptide, found predominantly in sensory C and A*δ* nerve fibers. It is a well-known very potent vasodilator of the skin and microvasculature [90] and plays an important role in neurogenic vasodilation of the skin [91]. It can potentiate both acetylcholine-mediated vasodilation and sweating [92]. CGRP has cardiovascular effects, proinflammatory actions, and metabolic effects [93] and often coexists with other peptides in sensory afferents, for example, substance P (SP), cholecystokinin, and dynorphin. Studies indicate that CGRP possibly plays a role in the transmission of nociception in the rat spinal cord, but the exact interactions with other nociceptive neurotransmitters in the spinal cord, such as SP, glutamate, and opioids are unknown [94]. Neuropeptides in the skin are synthesised and released predominantly by a subpopulation of small unmyelinated afferent neurons (C-fibers) designated as C-polymodal nociceptors, which represent about 70% of all cutaneous C-fibres and, to a far smaller extent, by small myelinated A*δ*-fibres [95]. Two forms of CGRP have thus far been isolated, CGRP-*α* and CGRP-*β*. CGRP-*α* occurs primarily in sensory neurons, whereas enteric neurons mainly contain CGRP-*β*. CGRP-*α* and CGRP-*β* are suggested to be regulated differently, and they probably act through different receptor subtypes [94]. Two receptor subtypes, CGRP1 and CGRP2, have been identified that are specific plasma membrane receptors. These are G-protein coupled and are able to activate adenylate cyclase and increase in intracellular cAMP that are sufficient to explain many of their effects [94, 96]. Other effects are NO dependent [97]. 

A wide distribution of CGRP messenger RNA, CGRP immunoreactive (IR) cell bodies, and nerve fibers is seen in the central nervous systems (CNS) of various species including the rat, cat, and human. CGRP-positive cells are also found in various autonomic ganglia but to a lesser extent in sympathetic principal neurones in the stellate and lumbar sympathetic ganglia. Some of the neurones, which contain both CGRP and vasoactive intestinal peptide (VIP), project to the sweat glands in rats [94, 98, 99]. 

CGRP fiber terminals are heavily concentrated in the dorsal horn of the rat spinal cord. The CGRP-containing axons are largely unmyelinated or small diameter myelinated fibres and constitute almost 30% of the primary afferent axons of the major afferent input to the superficial laminae of the dorsal horn [94]. It has been concluded that highly concentrated CGRP in nerve terminals is supplied by axonal transport from the neurone cell bodies [100]. 

### 4.1. CGRP and the Cardiovascular System

Microinjections of CGRP into the central nucleus of the amygdala elicited an increase in arterial blood pressure and heart rate in the rat [94]. In rats, low-intensity spinal cord stimulation induces cutaneous vasodilation that is possibly mediated by peripheral release of CGRP [101], which also increases the heart rate and force of contraction of the heart [94]. In humans, exogenously administered human *α*-CGRP showed vasodilatory action in the skin [25]. The vasodilation induced by CGRP may be achieved through more than one mechanism. In some tissues vasodilation correlates strongly with a rise in cAMP that is independent of nitric oxide (NO). In contrast, in other tissues (e.g., rat aorta), the effect is suggested to be NO-dependent via an NO-induced increase in cGMP [97]. In microvascular dermal endothelial cells, CGRP and SP have been shown to induce the release of NO [102]. K^+^ channels in arterial smooth muscle cells of rabbits are sometimes involved in CGRP-mediated vasodilation [94]. Hence, CGRP can activate various transduction signalling pathways and the vasodilation involves multiple second messengers [94].

### 4.2. CGRP and Sweat Glands

In the eccrine sweat glands, Zancanaro et al. [103] have found immunoreactivity for CGRP in secretory cells, granulated cells, and to some extent parietal cells. Immunoactivity of CGRP has also been detected in human axons of sudomotor cholinergic nerves stimulating eccrine sweat glands [104] where vasoactive intestinal peptide (VIP) has been shown to coexist [99, 104]. It has previously been reported that CGRP and VIP exert an influence on human sweating under physiological conditions [101]. It was therefore suggested that CGRP-(and SP-) containing neurones are involved in the local vasodilation associated with increased sweat production [103]. Immunoreactivity for NO was seen in myoepithelial cells (i.e., contractile cells within the sweat glands). The presence of CGRP, SP, and NO suggests local function interactions involving NO release, myoepithelial cell contraction, and vasodilation in the sweat glands [103].

### 4.3. CGRP and the Thermoregulatory Center

CGRP, when injected into the hypothalamus, can increase body temperature [105–108]. A recent study has shown that it decreases the rate of firing of warm-sensitive neurons in the thermoregulatory center, leading to hyperthermia [109]. It is possible that both central and peripheral actions are relevant for hot flushes [110].

### 4.4. Relationship between Estrogens, CGRP, and the Opioid System

CGRP-IR fibers have been observed in the superficial layers of the spinal dorsal horn with lower numbers of fibers in the deeper laminae of the spinal cord [111], the same location where Blomqvist et al. [112] found colocalisation of estrogen receptor IR and preproenkephalin messenger RNA expression. Estrogen injected subcutaneously in ovariectomized female rats results in a rapid increase in spinal cord enkephalin mRNA levels [113]. This suggests that estrogen influences opioid/enkephalin expression in the lower medulla and spinal cord (preferentially in the superficial layers) and may thereby exert a modulatory effect on sensory and nociceptive processing directly at the spinal and medullary levels [112] especially of relevance when studying pain. 

Endogenous and exogenous opioids modulate the release of the potent vasodilator CGRP at the spinal cord level [114, 115]. Gonadal hormones influence the endogenous opioid system [116]. In male castrated rats messenger RNA for the opioid precursor pro-opiomelanocortin is increased in the hypothalamus following testosterone and estradiol supplementation [117]. It has also been shown that there is a positive correlation between cerebrospinal *β*-endorphin concentrations and estrogen concentrations in plasma [118], and estrogen affects hypothalamic *β*-endorphin activity in rats [119].

In ovariectomised rats treated with estrogen (implanted silastic capsule), CGRP immunoreactivity and methionine-enkephalin immunoreactivity increased in the medial preoptic nucleus and the periventricular preoptic nucleus of the hypothalamus [120]. These findings show a connection between estrogens, enkephalins, and CGRP in the central nervous system very close to the thermoregulatory center. Thus, estrogens may affect CGRP production and release both directly and indirectly via opioids.

## 5. Acupuncture and CGRP

Since CGRP, like other neuropeptides, has a short half-life in the circulation system [121] and is degraded by neutral endopeptidase, tryptase, and chymase [122–124], much of its action cannot be measured in the blood, because degradation products circulate in the blood. However, we suggest that 24-hour urinary measurement of CGRP is a more reliable measurement of total amount of CGRP released into the circulation. Wyon and coworkers found a higher 24 hour urinary excretion of CGRP in women with vasomotor symptoms compared to excretion observed three months later after successful acupuncture therapy [22]. Twenty-four-hour CGRP excretion in urine was higher in postmenopausal women with flashes than in postmenopausal women without flashes and in fertile women [30]. In contrast, in men treated by castration due to prostate cancer, no changes were seen in urinary 24-hour excretion of CGRP three months after castration compared to before castration, neither in the group as a whole nor in the men who developed hot flashes [125]. Men treated successfully for hot flushes with acupuncture did not display a change in their 24 h urine CGRP excretion statistically [53]. 

Borud and co-workers could not find changes in CGRP excretion during acupuncture therapy of hot flashes, but this study included no 24-hour measurements of CGRP and was therefore not really suitable to answer the question if acupuncture therapy for hot flashes affects CGRP [126].

## 6. Conclusions and Suggestions for the Future

The effect of acupuncture on hot flashes in women and men [22, 48–53, 84] is probably multifactorial, and placebo-controlled studies are difficult to achieve, since there are so many components to be controlled for [75]. In the studies on acupuncture and hot flashes, efforts have been made to control the needling components, but in most cases no difference is seen between treatment groups whereas the within-group changes are evident [22, 53]. According to the hypothesis of the mechanisms of hot flashes, any intervention that increases estrogen, endorphin, or serotonin concentrations or decreases noradrenalin activity may be expected to reduce hot flashes in menopausal women. Exogenous estrogen seems to have an effect on many systems [31], probably affecting all neurotransmitters involved. Alternative treatments for hot flashes may affect one or perhaps several but not all of the systems involved; that is, acupuncture affects the *β*-endorphin levels [62] and also affects serotonin and noradrenalin activity in the central nervous system [70, 71]. Theoretically, by combining different alternative treatments, for example, SSRI and acupuncture, a synergistic effect of these treatments would appear with a better effect on hot flashes than any single treatment alternative would have by itself—except from that induced by estrogen treatment. Randomised studies are required to investigate this. 

Whether or not acupuncture has a direct effect on the release of CGRP in peripheral nerve endings remains to be investigated, and it is possible that other neurotransmitters, such as substance P, neurokinin A, neuropeptide Y, and adrenomedullin [110], are also involved in the pathogenesis of hot flashes. However, there is evidence that CGRP is involved in hot flashes in women and men with prostate cancer [11, 26–29], and a suggested treatment could therefore include a CGRP antagonist. There are a number of CGRP receptor antagonists in various stages of preclinical or clinical development, all of which are intended to treat acute episodes of migraine [127, 128]. Hopefully, these may become available in the future and tried as treatment alternatives for hot flashes. 

Recently, accumulating neuroimaging studies of humans have shown that acupuncture can modulate a widely distributed brain network. For example, the hypothalamus presented saliently intermittent activations during an fMRI session, both during needling and a prolonged period thereafter [129]. These new techniques may in the future be able to measure activity during hot flashes and perhaps also make it possible to study neurotransmitters involved in this process. 

What the real effect is of the needling component of acupuncture on hot flashes remains to be investigated. More randomised trials are needed if this is to be investigated satisfactorily, and in these not only the needling effect must be controlled for but also for the nonneedling components as well as for other nonspecific components as described by Langevin et al. [75].
